# Differentiated effects of social participation components on suicidal ideation across age groups in South Korea

**DOI:** 10.1186/1471-2458-13-890

**Published:** 2013-09-25

**Authors:** Chaelin Karen Ra, Youngtae Cho

**Affiliations:** 1 Institute of Health and Environment, Seoul National University, 1 Kwanak-ro, Kwanak-gu, Seoul, South Korea; 2 School of Public Health, Seoul National University, 1 Kwanak-ro, Kwanak-gu, Seoul, South Korea

**Keywords:** Social participation, Suicidal ideation, Age, Korean

## Abstract

**Background:**

Suicide among adults in the Korean population merits study to improve the understanding of the salient risk and protective factors because suicide rates in Korea have increased dramatically over the past 20 years. However, the association between social participation and suicidal ideation is poorly understood. Thus, this study aimed to identify the components of social participation in Korean society and to examine the processes through which the components of social participation influence the degree of suicidal ideation people experience across age groups.

**Methods:**

This study used survey data from the 2010 Seoul Welfare Panel Study. The sample population was restricted to adults aged 20 or older and was categorised into three groups by respondents’ ages. The groups were defined as 'young adults’ (aged 20–39), 'middle-aged adults’ (aged 40–64) and 'the elderly’ (age 65 or more). Three dimensions of social participation were identified by factor analysis – friendship network and hobby group, religious involvement, and instrumental social participation.

**Results:**

In the young adult group, only instrumental participation was statistically significant (-0.10, p = 0.06). In the middle-aged adult group, only friendship network and hobby group had a strong association with suicidal ideation (-0.11, p = 0.01). Interestingly, for the elderly, religious involvement was related to suicidal ideation, but in a positive way (0.26, p = 0.02).

**Conclusion:**

The study results supported the theory that different components of social participation are associated with a lower risk of suicidal ideation in different stages of adulthood.

## Background

Suicide rates in Korea have increased dramatically while at the same time declining, on average, in most of the Organisation for Economic Cooperation and Development (OECD) member countries. In Korea, deaths from suicide in 2009 were more than double the number reported in 1999 and were the highest among the 34 member countries of the OECD [1]. The number of suicides in South Korea hit 15,413 in 2009, which means that more than 40 South Koreans per day were taking their own lives. According to Statistics Korea (2011), suicide has become the number one cause of death in the country for those between 10 and 40 years of age, far above the number of those killed in traffic accidents or died from cancer [2]. Additionally, the suicide rate among the elderly has surged, increasing more than five times in the past 20 years [2]. Therefore, it is important that suicide among adults in the Korean population is studied to better understand the salient protective factors as well as the risk factors of suicide.

As mentioned above, suicide is a major social and public health problem in South Korea. Suicide is an example of an individual behaviour influenced by social integration, the extent to which the people in a society are bound together in social networks [3,4]. Thus, not only individual factors but also a range of social factors should be considered in attempts to explain suicide and suicidal behaviour [4].

A number of studies have mainly focused on completed suicide and suicide attempts [4]. Yet, there has been growing recognition of suicidal ideation as an important process in suicidal actions [4]. Suicidal ideation precedes suicide planning, which may result in an attempt leading to death [5]. Thus, ideation should be understood as an important phase in the suicide process, preceding suicide attempts and completed suicide [6].

Suicidal ideation shares many risk factors with suicidal attempts and completed suicides in the general population [7]. The risk factors include mood disorders, in particular, depression and its signs such as a continually depressed mood in adults [8]. Further, a low level of social support, low self-esteem and negative life events are reported to be the risk factors for suicidal ideation [8]. Other correlates include lack of reasons for living, higher than average life stress, relationship, low income, marital status, unemployment, lower level of hope and poor self-perceived health [4,7,8]. On the other hand, protective factors of suicidal behaviour in the general population include social support, self-appraisals, self-continuity, drawing on religious and moral beliefs, connecting with family and peers, a sense of belonging, and a sense of coherence [4,7,8]. Thus, it is not difficult to infer that a few of the protective factors can be driven from social participation.

Social participation is generally defined as a socially-oriented sharing of individual resources and has long been considered an important factor in an index of social integration [9]. Social participation is membership in a group or interaction with it. It leads to a sense of strength for the individual and brings broader emotional support and community involvement to the individual [4,10]. In previous studies, social participation has been shown to have protective health effects [11], including better self-rated health [12], a lower risk of death [13,14], healthier behaviours and better mental health, including well-being and happiness [9] and reduced distress [15]. Whereas there is a growing body of research on social participation and health, there are, to our knowledge, only a few studies examining the relationship between social participation and suicide. Durkheim’s early work suggests that higher levels of social participation are associated with reduced levels of suicide [3]. Later, Duberstein and his colleagues (2004) demonstrated that there is a relationship between suicidal tendencies, interaction with a social network and engagement in community activities [16]. It was concluded that the association between suicide and social/community indicators of poor social integration, which is mostly measured as social participation, is robust and largely independent of the presence of mental disorders [16]. In Korea, a recent study explored the relationship of social participation and suicidal ideation, and concluded that social participation acts as a mediator between social isolation and suicidal ideation [17].

As mentioned above, this focus on social participation and suicide is not new. However, previous studies rarely examined the effects of age on social participation; rather, they have primarily focused on elderly adults [12,17-21], or on a specific social activity such as religious service attendance [14], volunteer obligations [22] or participation in clubs [23].

Social participation is a multidimensional construct. Defining the components of social participation has varied in each study and differs across social contexts [18,20,24-27]. Hong and his colleagues (2003) mentioned that Koreans tend to participate more in hobby or culture groups and prefer participating in informal meetings to participating in public events [27]. Recent trends in Korea show that more people participate in gatherings through internet networking and that the traditional family/relative networks have weakened [27]. However, these concepts have never been empirically tested in the Korean context. Moreover, it is known that social participation changes as people age because of life transitions [20]. Older adults generally have limited opportunities to participate in social activities when compared with young adults and may experience relative deprivation [28]. Thus, the influence of social participation on health may differ among age groups.

The above considerations lead to the primary hypothesis of this research: the higher the extent of social participation, the lower the likelihood of having experienced suicidal ideation by an individual. This study aims first to identify the components of social participation in Korean society and then to examine the processes through which the components of social participation influence the degree of suicidal ideation people experience in different age groups.

## Methods

### Data

This study is a secondary analysis of cross-sectional data, using the survey data of the 2010 Seoul Welfare Panel Study (SWPS Wave 2) conducted by the Seoul Welfare Foundation. The SWPS is a publicly released dataset that is available at the website of the Seoul Welfare Panel Study (http://panel.welfare.seoul.kr). This research received IRB exemption from the IRB committee at the Graduate School of Public Health, Seoul National University, since its regulations clarified that studies used government administered publicly available secondary data were exempt from the review. The SWPS is a bi-annual longitudinal panel survey that began in 2008. It consists of a representative sample of households located in 25 administrative-areas in Seoul, South Korea. We used only Wave 2, as it contains the appropriate variables needed for the current study. Based on the number of respondents continuing from Wave 1, the response rate in Wave 2 for household members was 87.5%. More details of the survey design and methods are available at the above mentioned URL.

The sample population for these analyses was restricted to adults aged 20 or older, and the aggregate dataset was categorised into three groups by respondents’ ages. There were 5836 respondents in this analysis. The groups were defined as 'young adults’ (aged 20–39), 'middle-aged adults’ (aged 40–64) and 'the elderly’ (aged 65 or older). There were 1891, 2530 and 1415 respondents in each group accordingly. To improve the quality of data, we deleted missing data values.

### Measures

#### Suicidal ideation

Suicidal ideation was assessed by asking respondents the following question: 'Have you considered committing suicide in the past 12 months?’ This question had the three following response categories: once or twice, three times or more, or never. Before the statistical analyses, the responses were divided into never (0) and affirmative responses at any frequency (1).

#### Social participation

We applied factor analysis to clarify the nature of social participation because we were dealing with multiple related participation items and were trying to determine whether they formed one or more dimensions. The indicators of social participation were measured by 11 items, representing different aspects of social participation. Each item was assessed by the question, “How often do you participate in these activities?” and scored from 1 (rarely) to 5 (very often). Principal components analysis with varimax rotation was then performed in order to assess the components of social participation in Korea. Using the eigenvalue criterion (eigenvalue > 1) to determine the number of factors led to a two-factor-construct. After carefully examining a scree plot of the eigenvalues, we decided to investigate a three-factor-structure (Table 1). We identified three dimensions of social participation – friendship network and hobby group, religious involvement and instrumental social participation. The standardised Cronbach’s coefficient alpha was 0.91.

**Table 1:** Results of factor analysis*

	**Rotated factor loadings and communalities**		
**Items**	**Factor 1**	**Factor 2**	**Communality**
1. School reunion meetings	0.19	0.82	0.71
2. Sports and hobbies group	0.18	0.78	0.64
3. Hometown clubs (communities)	0.28	0.76	0.65
4. Family reunion	0.34	0.72	0.63
5. Religious involvement	0.24	0.45	0.26
6. Membership in not-for-profit organisations	0.75	0.36	0.69
7. Support for NGOs and arts	0.83	0.33	0.80
8. Educational board and committee meetings	0.89	0.26	0.85
9. Community meetings	0.75	0.18	0.60
10. Political party support	0.90	0.25	0.87
11. Membership in other organisations	0.70	0.30	0.59
*Variance*	4.23	3.07	7.29
*Cronbach’s α*	0.84	0.92	

*All parameter estimates are standardised.

Factor 1 – friendship network and hobby group.

Factor 2 – instrumental social participation.

Factor 3 – religious involvement.

#### Demographics

Survey items consisted of demographic characteristics including sex, marital status, educational attainment, household income, and employment status. Current marital status was recorded as 'never married’, 'currently married’ and 'other’. Educational attainment was divided into the following categories: 'less than high school graduation’, 'high school graduation’ and 'college or more’. Household income was recorded as one of four categories (1st to 4th quartiles). Employment status was dichotomised as 'currently employed’ and 'currently unemployed’.

#### Risk factors – stress and depression

The Perceived Stress Scale (PSS), the most widely used psychological instrument, was included in the SWPS 2010 [29]. The PSS was used to measure the degree to which situations in one’s life are perceived as stressful. It includes 10 items scored from 10 to 50. The SWPS 2010 included the Zung Self-Rating Depression Scale (ZSDS), one of the most frequently used self-administered scales for measuring depression. The ZSDS [30] consists of 20 items with four response choices ranging from none, some of the time, most, or all of the time, which are assigned numerical values from 1 to 4.

#### Protective factors

Several variables were included to measure protective factors of suicidal ideation. First, health behaviours were assessed by three variables: regular physical activity, current smoking status and alcohol consumption. Regular physical activity was measured by the responses (yes or no) for the question, 'During the last 7 days, have you done any moderate physical activity in your free time for at least 10 minutes?’ Current smoking status was coded as 'current smoker’ or 'non-smoker’ (including both 'never smoker’ and 'former smoker’). Drinking status was classified into two types (i.e., 'non-drinker’ and 'drinker’).

Leisure was measured by the sum of non-compulsory activities such as hobbies, watching television or listening to the radio, socialising with friends or family, attending cultural events, hosting events, and practising a sporting activity. The SWPS 2010 also included the Lubben Social Network Scale-6 (LSNS-6), a self-reported scale, to assess a respondent’s current social network by measuring perceived social support received by family and friends [31]. It consists of 6 items scored from 6 to 30. The Rosenberg Self-Esteem Scale (SES), the most widely-used self-esteem measure, was included [32]. The 10 items were answered on a five-point Likert scale ranging from 'strongly agree’ to 'strong disagree’.

### Statistical analysis

All analyses were performed using SAS version 9.2 to account for sampling design and to obtain proper variance estimation. First, an explanatory factor analysis was conducted. Next, using the factors identified as independent variables, logistic regression was used to investigate the probabilities of having experienced suicidal ideation for each age group.

## Results

Table 2 presents the unweighted frequency and weighted percentage distributions of all independent and controlling variables for the three age groups – young adults (aged 20–39), middle-aged adults (aged 40–64) and the elderly (aged 65 and over). There were no notable differences in the proportions of participation in the three social participation factors (friendship network and hobby group, religious involvement and instrumental social participation) between the age groups. Descriptive results of suicidal ideation are also included in Table 2. The proportion of adults reporting having experienced suicidal ideation tended to increase as the participants aged (3.66% in young adults, 5.1% in middle-aged adults and 6.88% in the elderly), consistent with previous findings.

**Table 2:** Sociodemographic characteristics of sample groups - Unweighted frequency and weighted proportion

	**Age groups**					
	**Young adults**		**Middle-aged adults**		**The elderly**	
	**N**	**%**	**N**	**%**	**N**	**%**
**Sex**						
Male	894	47.26	1137	44.88	589	41.68
Female	997	52.74	1393	55.12	826	58.32
**Marital status**						
Never married	932	50.28	122	4.33	12	0.64
Currently married	931	48.27	2137	86.23	889	64.16
Other	28	1.45	271	9.44	514	35.20
**Household income**						
1st percentile	216	11.62	468	17.46	773	52.11
2nd percentile	474	24.93	613	24.38	334	24.26
3rd percentile	611	31.25	700	28.30	201	14.93
4th percentile	590	32.20	749	29.86	107	8.70
**Educational attainment**						
Less than high school graduation	29	2.18	567	23.46	915	6.33
High school graduation	398	20.74	986	38.04	292	20.98
College or more	1464	77.08	977	38.49	208	14.69
**Employment status**						
Currently employed	1006	52.89	1415	55.48	195	14.21
Currently unemployed	885	47.11	1115	44.52	1220	85.79
**Regular physical activity**						
Sedentary	1231	64.44	1373	53.80	806	56.39
Regular or some	660	35.56	1157	46.20	609	43.61
**Smoking status**						
Non-smoker	1494	78.73	1999	79.20	1272	90.21
Current smoker	397	21.27	531	20.80	143	9.79
**Drinking status**						
Non-drinker	781	41.86	1326	52.10	1103	78.18
Drinker	1110	58.14	1204	47.90	312	21.82
**Leisure(M/SD)**	21.69	7.03	18.06	6.47	14.02	3.93
**Self-esteem(M/SD)**	33.32	4.72	33.10	4.64	30.99	4.48
**Social support(M/SD)**	19.16	1.56	18.98	1.60	18.67	1.61
**Social participation**						
Friendship network and hobby group(M/SD)	8.16	3.05	8.67	3.48	6.94	3.33
Instrumental social participation(M/SD)	9.36	3.94	9.59	4.16	7.69	2.98
Religious involvement(M/SD)	1.92	1.10	2.06	1.17	1.91	1.23
**Stress(M/SD)**	25.04	4.06	24.91	4.13	24.49	4.26
**Depression(M/SD)**	37.51	7.91	39.21	8.21	44.33	8.03
**Suicidal ideation**						
Never	1820	96.34	2406	94.9	1316	93.12
At least once in last 1 year	71	3.66	124	5.10	99	6.88
***Unweighted N***	**1891**		**2530**		**1415**	

A striking feature was the role of social participation, which was significant for all groups, but in different components when sex, marital status, household income, educational attainment, employment status, regular physical activity, smoking status, drinking status, leisure, self-esteem, social support, stress, depression and the three social participation factors were controlled for in the multiple logistic model (Table 3). In the young adult group, only instrumental participation was statistically significant (-0.10, p = 0.06). In the middle-aged adults group, only friendship network and hobby group had a strong association with suicidal ideation (-0.11, p = 0.01). Interestingly, in the elderly group, religious involvement was related to suicidal ideation, but in a positive way (positive association with suicidal ideation means an increase in suicidal ideation; 0.26, p = 0.02). This will be discussed further in the discussion.

**Table 3:** Adjusted coefficients of suicidal ideation across age groups

	**Age groups**					
	**Young adults**		**Middle-aged adults**		**The elderly**	
	**β**	**P-value**	**β**	**P-value**	**β**	**P-value**
**Social participation**						
Friendship network and hobby group	-0.02	0.79	-0.11	0.01	-0.06	0.40
Instrumental social participation	-0.10	0.06	-0.02	0.68	0.03	0.57
Religious involvement	0.12	0.40	-0.05	0.65	0.26	0.02
**Sex[Male]**						
Female	0.81	0.02	0.45	0.10	-0.74	0.02
**Marital status[Never married]**						
Currently married	0.21	0.74	0.94	0.03	-0.07	0.95
Other	-1.00	0.40	0.47	0.44	0.39	0.75
**Household income[1st percentile]**						
2nd percentile	-0.53	0.22	-0.12	0.68	0.04	0.89
3rd percentile	-1.04	0.03	-0.42	0.17	-0.92	0.06
4th percentile	-0.33	0.44	-0.39	0.25	0.39	0.40
**Educational attainment[Less than high school graduation]**						
High school graduation	-0.33	0.67	0.65	0.02	-0.17	0.63
College or more	-0.48	0.54	0.50	0.13	0.01	0.98
**Employment status[Currently employed]**						
Currently unemployed	0.36	0.22	-0.07	0.77	-0.88	0.08
**Regular physical activity[Sedentary]**						
Regular or some	0.18	0.53	-0.61	0.01	-0.27	0.33
**Smoking status[Non-smoker]**						
Smoker	0.14	0.73	0.01	0.97	-0.08	0.84
**Drinking status[Non-drinker]**						
Drinker	0.26	0.38	0.39	0.08	0.36	0.29
**Leisure**	0.01	0.56	0.01	0.44	-0.09	0.10
**Self-esteem**	-0.03	0.39	0.07	0.01	-0.02	0.62
**Social support**	0.15	0.08	-0.13	0.04	0.09	0.26
**Stress**	0.10	0.01	0.08	0.00	0.06	0.02
**Depression**	0.17	<.0001	0.14	<.0001	0.15	<.0001

Both stress and depression had positive associations with suicidal ideation in all age groups and were statistically significant (0.01 in young adults, 0.001 in middle-aged adults and 0.02 in the elderly for stress, and less than 0.0001 in all three groups for depression). Six variables (regular physical activity, smoking status, drinking status, leisure, self-esteem, social support) were tested to explore the relationships between protective factors and suicidal ideation. Regular physical activity and social support were independently and significantly related to suicidal ideation in middle-aged adults (-0.61, p < 0.01 and -0.13, p = 0.04, respectively).

## Discussion

The aim of this study was to investigate the differentiated effects of social participation components on suicidal ideation across age groups. Overall, it has been robustly demonstrated that social participation is associated with suicidal ideation across adulthood; however, the patterns of social participation constantly change. Three patterns of social participation were identified – instrumental social participation, participation in personal networks and hobby groups, and religious involvement. In this study, only instrumental social participation was significantly associated with low suicidal ideation among young adults, whereas only friendship network and hobby group had a negative association with suicidal ideation in middle-aged adults. For the elderly, religious involvement was positively related to suicidal ideation, which is inconsistent with general expectations.

As previously stated, it has been strongly demonstrated that social participation is associated with a lower risk of suicidal ideation. Social participation not only affects the positive rewards or feelings accumulated by the individual, but also leads to a sense of strength in the individual and brings the emotional support that people desire [9,28]. Therefore, the psychophysiological consequences of positive social participation may serve to decrease the risk of suicide as well as the risks of suicidal ideation and behaviour [3,17,33,34].

Then, why do the components of social participation affect the suicidal ideation differently across age groups in Korea? The individual is learning what the real world is, how it works, and his or her place in it in the earlier ages of adult life [35] and, jobs and marriages are undoubtedly considered as primary goals at this stage. However, through the economic crisis in the late 1990s and the late 2000s, it became more difficult for young people to find jobs in Korea [36]. Many young adults are hired as temporary or part-time workers at a low pay rate, making them unable to afford to get married or have children [37]. They are Korea’s most highly-educated generation (65% of young adults aged 25–34 received college education or higher), yet their unemployment rate is at its highest level in South Korea [38]. Indeed, many young people are dissatisfied with political structures that are unresponsive to their needs and interests, but that they remain interested in social and political issues and continue to seek recognition from the political system [39]. Thus, it is not difficult to infer that young adults may gain positive rewards or feel self-worth through participating in instrumental participation such as private-party support or community meetings as they can raise their voices together for change. As a result, instrumental participation may be more valuable than other types of social participation for young adults in Korea. Hence, those with active instrumental participation tend to show lower suicidal ideation than others.

During midlife, individuals experience major life events such as empty nest, menopause, retirement and death of parents [40,41]. Particularly, Korean middle-aged adults today have been affected by socio-cultural factors such as economic downturn and an aging society [38]. Midlife in Korean society is now undoubtedly a phase of heavier responsibilities, for both men and women, in work and family areas than ever before. They are expected to be at a peak in their career roles to provide for their families and to earn a sense of achievement for themselves whilst they are facing premature withdrawal from work, perceived conflict between career and family life, and perceived discrepancy between aspiration and achievement [38,41,42]. These responsibilities bring about feelings of loneliness in family life, feelings of low energy and weakening, anxiety over aging, and senses of loss and worthlessness [42]. Therefore, middle-aged individuals are careful to select activities that are personally and emotionally meaningful so that these feelings are resolved satisfactorily [43]. Middle aged adults prefer interactions with familiar or intimate social partners, usually close family members and close friends [35,43]. This explains why many middle-aged adults value participating in personal networks and hobby group activities such as school reunion meetings and hometown clubs. For instance, “Iloveschool,” an online Korean alumni service connects people who graduated from the same primary school in the same year and homeroom. It was very popular in the early-2000s, among middle-aged adults. It can be concluded that middle-aged adults obtain emotional support through smaller group participation. Consequently, in this age group, the more that people were involved in this type of social participation, the less that suicidal ideation was reported.

The elderly reported the highest level of positive emotional experiences when interacting with family members [44]. However, social support from family for the elderly has rapidly declined, particularly in urban areas such as Seoul [45]. Thus, it can easily be interpreted that the significance of other social support systems has been overly stressed and that the ability of the family to act as an informal caregiving institution needs to be critically reviewed [45]. However, these public systems are not well established yet, and instead, churches or other religious involvements function as “a pseudo-extended family” in Korean society [45]. In particular, they provide emotional support and a helping hand to those individual members who are psychologically distressed or experiencing other personal crises. The elderly are the ones who need support systems the most. Consequently, religious involvement in late adulthood is more related to suicidal ideation than the other types of social participation.

Interestingly, contrary to previous findings that religious involvement is negatively associated with suicidal ideation, the association between the two in this study was significantly positive. However, our preliminary research found that religious involvement was negatively related to suicidal ideation until depression was added as a control variable. This result suggests that a strong association between depression and suicidal ideation exists among the elderly. Therefore, it can be concluded that the depressed elderly are more likely to be involved in religion than the non-depressed elderly. A number of empirical studies support the obvious hypothesis that suicide is related to depression across ages, although the magnitude of the relationship between depression and suicide varies in each age group [46-48]. Depression may be the strongest predictor of future attempts of suicide among the elderly.

The present study has certain limitations that should be accounted for when considering the study and its contributions. The study sample was limited to the population of a metropolitan city. Thus, results of this study should be applied cautiously to the discussion of those residing in rural areas. Moreover, the causal directions of associations are difficult to discern and need to be studied further due to the cross-sectional nature of the datasets employed. Generalisation of the findings in this study should be carefully applied since the information on social participation in the data is not available elsewhere. Also, the components of social participation may not be the same in other countries.

## Conclusions

Despite these limitations, this study has a number of strengths. First, the current study analysed data from a large, representative sample in Seoul, Korea. Additionally, this was the first reported attempt to separate the components of social participation, and to examine the associations between different components of social participation and suicidal ideation across different age groups. Lastly, this study provides the first empirical evidence of the patterns of social participation in the unique Korean context.

## Competing interests

The authors declare that they have no competing interests.

## Authors’ contributions

CKR (first author) conducted analyses and wrote the first draft. YC (corresponding author) crafted research design and participated in the write-up of manuscript. Both authors read and approved the final manuscript.

## Pre-publication history

The pre-publication history for this paper can be accessed here:

http://www.biomedcentral.com/1471-2458/13/890/prepub
